# Dynamical Behavior of Disordered Regions in Disease-Related Proteins Revealed by Quasielastic Neutron Scattering

**DOI:** 10.3390/medicina58060795

**Published:** 2022-06-13

**Authors:** Satoru Fujiwara

**Affiliations:** Institute for Quantum Biology, National Institutes for Quantum Science and Technology, 2-4 Shirakata, Tokai, Ibaraki 319-1106, Japan; fujiwara.satoru@qst.go.jp

**Keywords:** quasielastic neutron scattering, protein dynamics, intrinsically disordered protein, synuclein, troponin, Parkinson’s disease, hypertrophic cardiomyopathy

## Abstract

*Background and Objectives*: Intrinsically disordered proteins (IDPs) and proteins containing intrinsically disordered regions (IDRs) are known to be involved in various human diseases. Since the IDPs/IDRs are fluctuating between many structural substrates, the dynamical behavior of the disease-related IDPs/IDRs needs to be characterized to elucidate the mechanisms of the pathogenesis of the diseases. As protein motions have a hierarchy ranging from local side-chain motions, through segmental motions of loops or disordered regions, to diffusive motions of entire molecules, segmental motions, as well as local motions, need to be characterized. *Materials and Methods*: Combined analysis of quasielastic neutron scattering (QENS) spectra with the structural data provides information on both the segmental motions and the local motions of the IDPs/IDRs. Here, this method is applied to re-analyze the QENS spectra of the troponin core domain (Tn-CD), various mutants of which cause the pathogenesis of familial cardiomyopathy (FCM), and α-synuclein (αSyn), amyloid fibril formation of which is closely related to the pathogenesis of Parkinson’s disease, collected in the previous studies. The dynamical behavior of wild-type Tn-CD, FCM-related mutant Tn-CD, and αSyn in the different propensity states for fibril formation is characterized. *Results*: In the Tn-CD, the behavior of the segmental motions is shown to be different between the wild type and the mutant. This difference is likely to arise from changes in the intramolecular interactions, which are suggested to be related to the functional aberration of the mutant Tn-CD. In αSyn, concerted enhancement of the segmental motions and the local motions is observed with an increased propensity for fibril formation, suggesting the importance of these motions in fibril formation. *Conclusions*: Characterization of the segmental motions as well as the local motions is thus useful for discussing how the changes in dynamical behavior caused by the disease-related mutations and/or environmental changes could be related to the functional and/or behavioral aberrations of these proteins.

## 1. Introduction

The basic concept underlying structural biology is that the structure defines the function. Since the structural determination of myoglobin [1] and hemoglobin [2], structures of numerous proteins have been determined mainly by X-ray crystallography, and structural biology contributes significantly to the advancement in the understanding of the molecular mechanisms of protein functions. During this huge success in structural biology, it was noticed that proteins often contain regions that cannot be observed by crystallography, but not much attention was paid to such disordered regions. However, growing evidence suggests that such intrinsically disordered regions (IDRs) and intrinsically disordered proteins (IDPs), which adopt no distinct tertiary structures but fluctuate many structural substrates, play important roles in protein functions [3,4,5,6]. It is now well accepted that they are common (44% of human-coding genes contain disordered regions that contain 30 or more amino acids in length) and involved with a variety of cellular functions [7]. In particular, the IDPs and IDRs participate in functions related to molecular recognition and control/regulation of signaling pathways through their ability to interact with many other proteins. This implies that misfolding of these regions may result in failure of proper interactions, thereby causing dysfunction of the related proteins, which could lead to the pathogenesis of related diseases. Indeed, with amyloid fibril formation in various diseases being the most notable example [8], various IDPs/IDRs have been shown to be involved in various human diseases [9]. For elucidation of the mechanisms of the pathogenesis of such diseases, it is important to elucidate the behavior of the IDPs/IDRs involved with the diseases. In particular, since the IDPs/IDRs are fluctuating between many structural substrates, characterization of the dynamical behavior is important.

The dynamical behavior of the IDPs/IDRs has been investigated using various biophysical techniques, including nuclear magnetic resonance (NMR) spectroscopy and fluorescent spectroscopy such as Förster resonance energy transfer (FRET), and neutron scattering spectroscopy such as incoherent quasielastic neutron scattering (QENS). NMR spectroscopy provides the information at the amino-acid residue level, and fluorescent spectroscopy such as FRET provides the information on the labeled residues. The information on the dynamics obtained by these techniques is, however, somewhat “indirect” because the parameters describing the dynamics rely on the assumptions specific to the techniques. On the other hand, neutron spectroscopy provides a unique tool for directly measuring the dynamics of proteins. The QENS signals arise primarily from incoherent neutron scattering of hydrogen (H) atoms, which reflects the motions of H atoms. The QENS measurements on proteins in D_2_O-solvent thus provide information on the motions of H atoms in proteins. Because the motions of H atoms reflect the motions of larger groups to which the H atoms are bound, and because H atoms are distributed (pseudo-)uniformly within proteins, the QENS spectra provide information on the average dynamics of the proteins [10].

Proteins in solution undergo a hierarchy of motions, ranging from local motions of side- and main-polypeptide chains, through segmental motions of loops and/or disordered regions and relative domain motions, to diffusive motions of the entire molecules, and these motions contribute to the QENS spectra of the solution samples of proteins in D_2_O solvent [11]. The QENS spectra of such samples are often analyzed using a phenomenological equation containing two Lorentzian functions so that the contributions from the local motions and those from the larger scale motions are distinguished (see below). We carried out the QENS measurements on the solution samples of hemoglobin [12], consisting of four subunits, the troponin core domain (Tn-CD) [13], containing IDRs, and α-synuclein (αSyn) [14], which is an IDP, and found that the contributions from the domain (subunit) motions as well as the segmental motions of the IDRs and the IDP could be significant. Thus, if the contributions of the diffusive motions of the entire molecules could be estimated, the contributions of the domain/segmental motions should be extracted. We have thus developed an analysis method that can extract motions at the segment/domain level by combining QENS, small-angle X-ray scattering (SAXS), and dynamic light scattering (DLS) measurements and demonstrated its feasibility [15]. The extraction of the segmental motions is critical for the characterization of the IDPs/IDRs. Previous studies on the Tn-CD [13] and αSyn [14] did not use this method, and thus the analysis of the segmental motions was qualitative. Here, we re-analyze the QENS spectra of these proteins by this method and characterize the behavior of the disordered regions in these proteins quantitatively.

Tn, consisting of three subunits, TnC, TnI, and TnT, is one of the major components of thin filaments in muscle. Together with other major proteins, actin, and tropomyosin (Tm), Tn plays a central role in the regulation of muscle contraction in a Ca^2+^-dependent manner [16]. When the concentration of Ca^2+^ increases in muscle cells, Ca^2+^ binds to TnC, and this triggers a series of structural changes in TnC, TnI, and TnT, which results in a conformational change in Tm from the state that another major protein in muscle, myosin, whose interaction with actin makes the muscle contract, cannot interact with actin to the state that myosin can interact with actin and thereby making muscles contract. The Tn-CD contains the central region of the pathway of the signal propagation of Ca^2+^ binding. Many mutations in this protein are known to be related to familial cardiomyopathy (FCM) [17], and how such mutations cause functional aberration of Tn, thereby leading to the pathogenesis of FCM, has been shown for various mutations [18]. Investigating how the behavior of the disordered regions in the Tn-CD changes by mutations could provide insights into the mechanism of the functional aberration of Tn.

αSyn is an IDP of 14 kDa, abundant in presynaptic nerve cells [19], and is involved with various functions related to synaptic plasticity, vesicle dynamics, and neurotransmitter release [20]. This protein is known to form amyloid fibrils, and this fibril formation is closely related to the pathogenesis of Parkinson’s disease [21]. Because αSyn is an IDP, the dynamical behavior of αSyn is directly related to the formation of fibrils. Since αSyn has a distinct propensity for fibril formation depending on the solution conditions, a comparison of the dynamical behavior of αSyn in different propensity states should provide insights into the mechanism of fibril formation and thus provide clues to elucidate the mechanism of the pathogenesis of Parkinson’s disease.

Here, we discuss through re-analysis of the QENS spectra of the two examples, the Tn-CD that contains the IDRs and αSyn that is an IDP, how the dynamical behavior of the disordered regions/disordered protein is related to the functional aberration or behavior that leads eventually to the pathogenesis of the diseases.

## 2. Materials and Methods

### 2.1. Materials

The QENS spectra analyzed here are those of the Tn-CD and αSyn. Since muscle contraction is regulated in a Ca^2+^-dependent manner, the QENS spectra of the Tn-CD were obtained in low- and high-Ca^2+^ states. The solution in the low-Ca^2+^ state, prepared in D_2_O, contained 50 mM HEPES (pD 8.0), 0.5 M NaCl, 5 mM MgCl_2_, and 5 mM ethylene glycol tetraacetic acid, whereas that in the high-Ca^2+^ state, prepared in D_2_O, contained 50 mM HEPES (pD 8.0), 0.5 M NaCl, 5 mM MgCl_2_, and 5 mM CaCl_2_ [13]. Protein concentrations in these solutions were ~22 mg/mL [13]. The low-Ca^2+^ state corresponds to the condition where muscle contraction does not occur, whereas in the high-Ca^2+^ state, muscle contraction occurs. The QENS spectra in the low- and high-Ca^2+^ states were measured on the wild-type Tn-CD (wtTn-CD) and the Tn-CD containing the mutation K247R in TnT (mtTn-CD) [13]. This mutant is found in patients with hypertrophic cardiomyopathy [22,23]. This mutation increases the maximum ATPase activity without changing Ca^2+^ sensitivity [24]. As this mutation is located in the middle of the signal-propagation pathway from TnC to Tm via TnT [25], understanding the behavior of this mutant should provide insights into the regulatory mechanism itself.

In the case of αSyn, the QENS spectra of this protein in the low-salt solution at neutral pH and the high-salt solution at neutral pH are analyzed [14]. Both solutions were prepared in D_2_O. The low-salt solution contained 20 mM HEPES (pD 7.4), whereas the high-salt solution contained 20 mM HEPES (pD 7.4) and 150 mM NaCl [14]. Protein concentrations in these solutions were ~10 mg/mL [14]. αSyn in the low- and high-salt solutions has a low and high propensity for fibril formation, respectively. A comparison of the dynamical behavior between these different propensity states should provide clues to specify the key behavior leading to the start of the process of fibril formation.

The QENS spectra analyzed here were obtained from the measurements using the near-backscattering spectrometer, BL02 (DNA) [26] at the Materials and Life Science Experimental Facility of the Japan Accelerator Research Complex, Japan, at the energy resolution of 12 μeV, at which atomic motions faster than 55 ps are accessible.

### 2.2. Methods

The QENS spectra of protein solutions, S(*Q,ω*), are often analyzed using a phenomenological equation containing two Lorentzian functions, one of which represents the global motions such as translational and rotational diffusion of the entire protein molecule as a rigid body, and the other of which represents the convolution of the global motions and the local motions such as the side-chain motions, as described in Equation (1) [11].

(1) $$S\left(Q,\omega \right)=\left[A_{0}\left(Q\right)L_{\mathrm{global}}\left(Q,\omega \right)+\left\{1-A_{0}\left(Q\right)\right\}L_{\mathrm{local}}\left(Q,\omega \right)⊗L_{\mathrm{global}}\left(Q,\omega \right)\right]⊗R\left(Q,\omega \right)+B\left(Q\right),$$

where *Q* (= 4πsin*θ*/*λ*, where 2*θ* is the scattering angle and *λ* is the wavelength of incident neutrons) is the momentum transfer, *ω* is the energy transfer between the incident and the scattered neutrons, which corresponds to the energy of the motions of the molecules, *A*_0_(*Q*) is the elastic incoherent structure factor (EISF), *L*_global_(*Q,ω*), and *L*_local_(*Q,ω*) denotes the Lorentzian functions representing the global and local motions, respectively, having the form of (1/π) × (Γ(*Q*)/(Γ(*Q*)^2^ + *ω*^2^), where Γ(*Q*) is the half-width at half-maximum of the Lorentzian function. *R*(*Q,ω*) is the instrumental resolution function, *B*(*Q*) is the background, and ⊗ denotes the convolution operation. Since the convolution of two Lorentzian functions is also a Lorentzian function, fitting the spectra with the equation containing the two Lorentzian functions distinguish the global motions and the local motions. Whereas *L*_global_(*Q,ω*) of well-folded globular proteins contains only the contributions from the diffusive motions as a rigid body, *L*_global_(*Q, ω*) of those containing the disordered regions contains a non-negligible contribution from the segmental motions [15]. In such cases, the QENS spectra can be described as a first approximation that translational and rotational diffusion as a rigid body and the segmental motions are independent of each other, as:

(2) $$\begin{array}{l} S\left(Q,\omega \right)=[A_{0}\left(Q\right)L_{\mathrm{trans}}\left(Q,\omega \right)⊗L_{\mathrm{rot}}\left(Q,\omega \right)⊗L_{\mathrm{seg}}\left(Q,\omega \right)\\ \;\;\;\;\;\;\;\;\;\;\;\;\;\;\;\;\;\;\;\;\;\;\;\;\;+\left\{1-A_{0}\left(Q\right)\right\}L_{\mathrm{trans}}\left(Q,\omega \right)⊗L_{\mathrm{rot}}\left(Q,\omega \right)⊗L_{\mathrm{seg}}\left(Q,\omega \right)⊗L_{\mathrm{local}}\left(Q,\omega \right)]⊗R\left(Q,\omega \right)+B\left(Q\right), \end{array}$$

where *L*_trans_(*Q*,*ω*), *L*_rot_(*Q*,*ω*), and *L*_seg_(*Q*,*ω*) represent the translational diffusion, the rotational diffusion, and the segmental motions, respectively. *L*_global_(*Q,ω*) in Equation (1) corresponds to the component *L*_trans_(*Q*,*ω*)⊗*L*_rot_(*Q*,*ω*)⊗*L*_seg_(*Q*,*ω*) in Equation (2). Thus, if the contribution of *L*_trans_(*Q*,*ω*)⊗*L*_rot_(*Q*,*ω*) (=S_trans+rot_(*Q*,*ω*)) can be estimated, *L*_seg_(*Q*,*ω*) can be extracted. S_trans+rot_(*Q*,*ω*) can be simulated if the structural models of the proteins are available. If the translational and rotational diffusion coefficients, D_T_ and D_R_, and the radial density distribution, *ρ*(*r*), are calculated from the structural models, S_trans+rot_(*Q*,*ω*) can be described as [27,28]:

(3) $$S_{\mathrm{trans}+\mathrm{rot}}\left(Q,\omega \right)=\frac{1}{\pi }\sum_{l=0}^{\infty }B_{l}\left(Q\right)\cdot \frac{D_{R}l\left(l+1\right)+D_{T}Q^{2}}{\omega^{2}+{\left(D_{R}l\left(l+1\right)+D_{T}Q^{2}\right)}^{2}},$$

with

(4) $$B_{l}\left(Q\right)=\int_{0}^{R_{\max}}\rho \left(r\right)\left(2l+1\right)j_{l}{}^{2}\left(Qr\right)dr,$$

where *R*_max_ is the maximum length from the origin in *ρ*(*r*), and *j_l_*(*Qr*) is the *l*-th order spherical Bessel function of the first kind.

**Figure 1:**
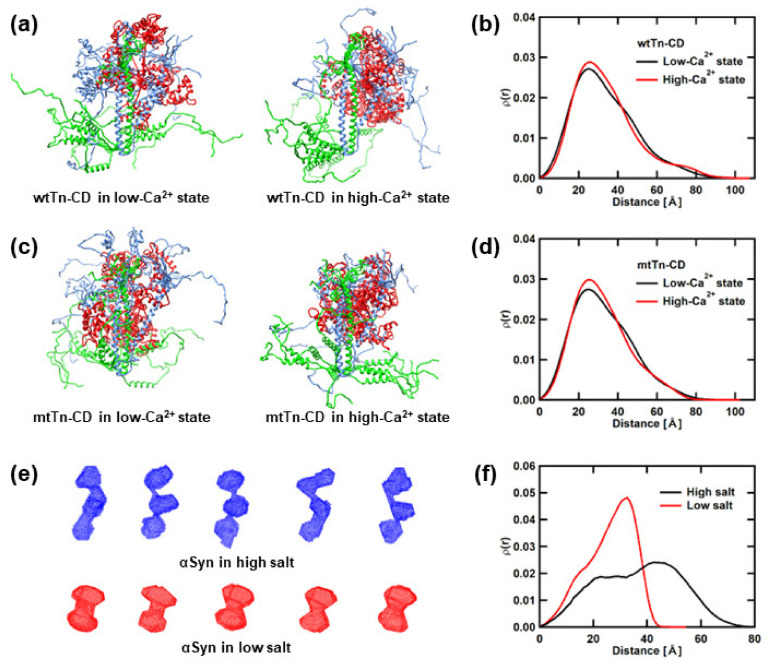
Summary of the structural models. (**a**,**c**) Gallery of examples of the structural models of the wtTn-CD and the mtTn-CD, respectively. A total of 10 models, arbitrarily selected from the ensembles of 1000 models, are superimposed so that the central regions observed by the crystal structure coincide. (**e**) Gallery of examples of the structural models of αSyn. A total of 5 examples of the dummy-atom models, arbitrarily chosen from the ensembles of 100 models, are shown. (**b**,**d**,**f**) the *ρ*(*r*) curves of the wtTn-CD, the mtTn-CD, and αSyn, respectively. Panels (**a**,**c**) are drawn using UCSF Chimera [36], and (**e**) are drawn using PyMOL.

The structural models of the proteins containing the IDRs and the IDPs must be ensembles of the structures as there are no definite, distinct structures. Here, as the structural models, ensembles of the structural models, calculated based on the SAXS curves (taken from [29] for the Tn-CD and [14] for αSyn), are employed. Figure 1a,c,e show examples of the structural models of the wtTn-CD, the mtTn-CD, and αSyn, respectively. The structural models of the Tn-CD were obtained by generating the atomic models, constructed by adding the missing residues to the crystal structure of the Tn-CD (PDB accession number: 4Y99) and searching for appropriate structures that provide model curves that are consistent with the experimental SAXS curves [29]. An ensemble of 1000 atomic models was generated for each condition. The structural models of αSyn, on the other hand, were obtained as the dummy-atom models from the analysis using the program DAMMIF [30] in the program suit ATSAS [31]. An ensemble of 100 models was generated for each condition. The D_T_ and D_R_ values of each model in the ensembles are calculated using the programs Hydropro [32] or Hydro++ [33], using the viscosity values of D_2_O [34], corrected for the presence of NaCl [35], and then averaged over all the models in the ensembles. The values of D_T_ and D_R_ obtained are summarized in Table 1. The *ρ*(*r*) curve is also calculated from each model in the ensembles and then averaged. Figure 1b,d,f show these curves of the wtTn-CD, the mtTn-CD, and αSyn, respectively. The simulated spectra corresponding to *S*_trans+rot_(*Q*,*ω*) are calculated using these averaged values and the curves, according to Equations (3) and (4). These simulated spectra are further approximated by the Lorentzian functions, the widths of which are used to estimate the values of D_trans+rot_ as described below. 

## 3. Results

### 3.1. Analysis of the QENS Spectra of the Tn-CD

**Figure 2:**
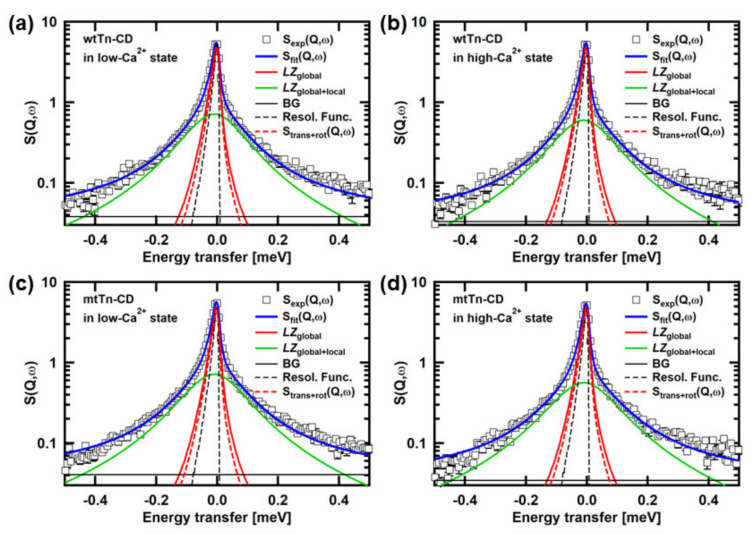
Examples of the QENS spectra. The spectra at *Q* = 1.05 Å^−1^ of (**a**) the wtTn-CD in the low-Ca^2+^ state, (**b**) the wtTn-CD in the high-Ca^2+^ state, (**c**) the mtTn-CD in the low-Ca^2+^ state, (**d**) the mtTn-CD in the high-Ca^2+^ state, are shown. The fits to the experimental spectra using Equation (1), the simulated spectra of S_trans+rot_(*Q*,*ω*), and the resolution function are also shown. The experimental spectra shown were obtained from the measurements carried out during the study described in [13].

Figure 2 shows examples of the QENS spectra of the Tn-CD. The spectra of the wtTn-CD and the mtTn-CD in the low- and high-Ca^2+^states are shown. The simulated curves of S_trans+rot_(*Q*,*ω*) are also shown, along with the fits by the two Lorentzian functions, denoted by *LZ*_global_ and *LZ*_global+local_, the former and the latter of which correspond to the first and the second terms in the square bracket in Equation (2), respectively. It is clearly shown that S_trans+rot_(*Q*,*ω*) and *LZ*_global_ have distinct widths, indicating that the contribution from the segmental motions is not negligible. The crystal structure of the Tn-CD (PDB accession number: 4Y99) shows several missing residues (the residues 86–90 in TnC, 183–198 and 272–288 in TnT, and 31–42, 138–147, and 167–210 in TnI). The contribution of the segmental motions is thus likely to reflect the motions of these residues, in particular, the motions of the terminal residues such as TnI_31–42_, TnI_167–210_, TnT_183_–_198_, and TnT_272–288_.

**Figure 3:**
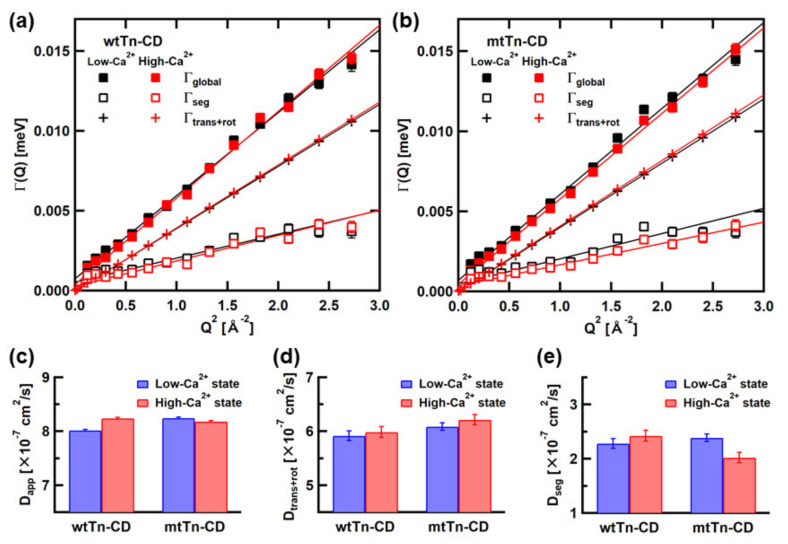
Summary of Q^2^-dependence of the Γ values of (**a**) the wtTn-CD and (**b**) the mtTn-CD, and summary of the diffusion coefficients, (**c**) D_app_, (**d**) D_trans+rot_, and (**e**) D_seg_, obtained from linear fits to the plots in (**a**,**b**). Error bars within the symbols are not shown.

Assuming that *L*_seg_(*Q*,*ω*) can be represented by the Lorentzian function, *L*_seg_(*Q*,*ω*) can be deconvoluted from *L*_global_(*Q,ω*), which is represented by the Lorentzian function corresponding to *LZ*_global_ in Figure 2, using the simulated S_trans+rot_(*Q*,*ω*). The widths of the Lorentzian functions, Γ_global_ and Γ_seg_, corresponding to *L*_global_ (*Q*,*ω*) and *L*_seg_(*Q*,*ω*), respectively, are plotted against Q^2^ in Figure 3a,b. The widths of the Lorentzian functions, Γ_trans+rot_, fit to S_trans+rot_(*Q*,*ω*) are also plotted. Slopes of linear fits to these Γ vs. Q^2^ plots provide “apparent” diffusion coefficients, assuming free diffusion. The slopes of the fits to *L*_global_(*Q*,*ω*), S_trans+rot_(*Q*,*ω*), and *L*_seg_(*Q*,*ω*) provide D_app_, D_trans+rot_, and D_seg_, respectively. D_app_ contains the contributions from translational and rotational diffusion of the entire molecule and the segment motions. D_trans+rot_, which is obtained from the linear fit to the widths of the Lorentzian functions, which fit the simulated S_trans+rot_(*Q*,*ω*), contains the contributions from translational and rotational diffusion of the entire molecule as a rigid body. D_seg_ contains the contribution from the segmental motions. The estimated values of D_app_, D_trans+rot_, and D_seg_ are shown in Figure 3c–e, respectively. These values are also summarized in Table 2. D_app_ obviously shows different behavior in response to [Ca^2+^] between the wild type and the mutant. As D_trans+rot_, which reflects the structure of the Tn-CD, shows similar behavior between the wild type and the mutant, this difference in the behavior arises from the difference in segmental motions. As shown in Figure 3e, the D_seg_ value of the wtTn-CD is larger in the high-Ca^2+^ state than in the low-Ca^2+^ state, whereas that of the mtTn-CD is lower in the high-Ca^2+^ state than in the low-Ca^2+^ state.

**Figure 4:**
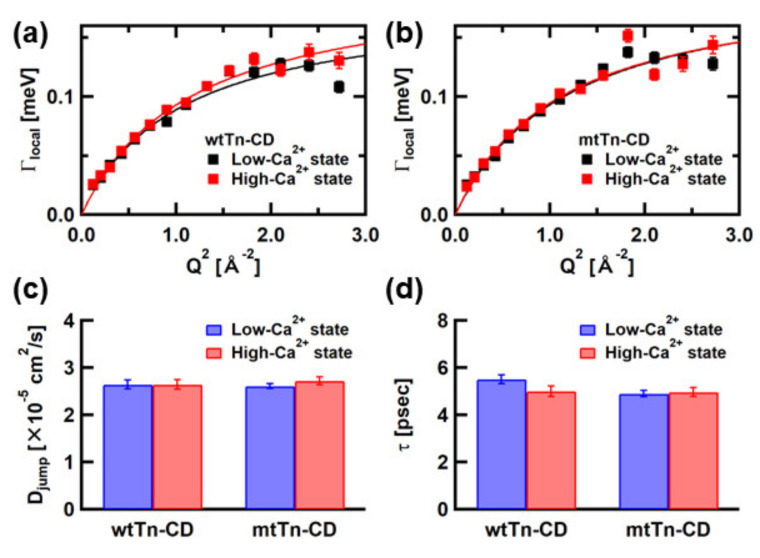
Summary of Q^2^-dependence of Γ_local_ of (**a**) the wtTn-CD and (**b**) the mtTn-CD, and summary of the parameters, (**c**) D_jump_, and (**d**) the residence time, obtained from the fits with the equation based on the jump-diffusion model to the plots in (**a**,**b**). Error bars within the symbols are not shown.

We also re-analyze *L*_local_(*Q*,*ω*), which reflects the local motions, such as the side-chain motions, to verify any possible correlation between these motions. Figure 4a,b show Q^2^-dependences of the widths, Γ_local_, of *L*_local_(*Q*,*ω*). These curves can be fitted with a function based on the jump-diffusion model [11], described as,

(5) $$\Gamma_{local}=\frac{D_{\mathrm{jump}}Q^{2}}{1+D_{\mathrm{jump}}Q^{2}\tau },$$

where D_jump_ denotes the jump-diffusion coefficient, and τ denotes the residence time. Figure 4c,d show the values of D_jump_ and τ. D_jump_ is virtually the same in the low- and high-Ca^2+^ states in both the wtTn-CD and the mtTn-CD. The residence time in the wtTn-CD is shorter in the high-Ca^2+^ state than in the low-Ca^2+^ state, whereas no change is observed in the mtTn-CD, suggesting that the local motions become faster in the wtTn-CD while they remain the same in the mtTn-CD.

**Figure 5:**
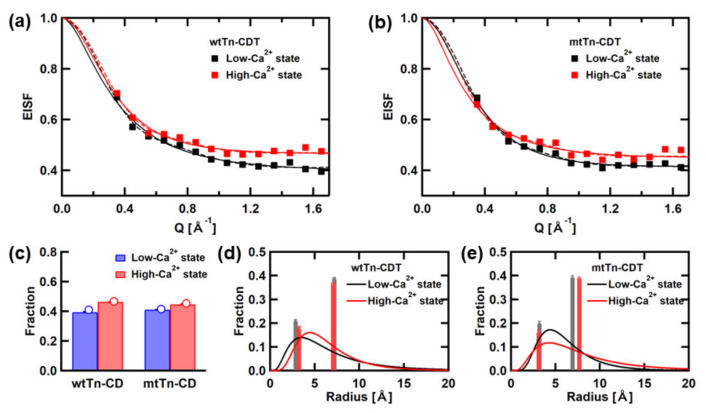
Summary of the EISF curves and their analysis using Equation (6). (**a**,**b**) The EISF curves of the wtTn-CD and the mtTn-CD, respectively. Solid lines are the results of the analysis using Equation (6). Dashed lines are the results of the analysis based on the diffusion-inside-a-sphere model with two spheres of distinct radii employed in the previous study [13]. (**c**) Summary of the fraction of the “immobile” atoms. Bars represent the results of the analysis using Equation (6). Open circles represent the results of the analysis based on the diffusion-inside-a-sphere model with two spheres of distinct radii (Equation (7)). (**d**,**e**) The radial distribution of the fraction of the “mobile” atoms in the wtTn-CD, and the mtTn-CD, respectively. Solid lines represent the results using Equation (6). Bars represent the results of the analysis using Equation (7).

Figure 5a,b show Q-dependences of the EISF (*A*_0_ in Equation (1)), from which the information on amplitudes of the local atomic motions can be obtained. These EISF curves are analyzed using the equation based on the assumption that diffusive motions of the atoms are confined in spherical space with radii that follow a log-normal distribution [37], described as,

(6) $$\mathrm{EISF}\left(Q\right)=p+\left(1-p\right)\int_{0}^{\infty }\frac{1}{s\sqrt{2\pi }a}e^{-\frac{{\left(\ln\left(a/c\right)\right)}^{2}}{2s^{2}}}{\left[\frac{3j_{1}\left(Qa\right)}{Qa}\right]}^{2}da,$$

where p is the fraction of atoms whose motions are outside the instrumental energy window and therefore appear “immobile”, (1 − p) corresponds to the fraction of atoms diffusing within the spherical spaces, *c* is the median of the distribution; *s* is the variance in the natural logarithmic space, and *j*_1_ denotes the first-order spherical Bessel function of the first kind. The results of the fits are also shown. The values of the fraction of the “immobile” atoms, p, are shown in Figure 5c, and the radial distributions of the mobile fractions are shown in Figure 5d,e. We also show the results of the EISF analysis using the diffusion-inside-a-sphere model [38], carried out in the previous study [13], for comparison in Figure 5. The equation based on the model containing two types of populations of atoms undergoing diffusive motions within spheres of different radii was employed to fit the EISF curves:

(7) $$\mathrm{EISF}\left(Q\right)=p_{0}+p_{1}\times {\left(3j_{1}\left(Qa_{1}\right)/Qa_{1}\right)}^{2}+p_{2}\times {\left(3j_{1}\left(Qa_{2}\right)/Qa_{2}\right)}^{2},$$

where p_0_ is the “immobile” fraction, and p_1_ and p_2_ are the fractions of atoms undergoing diffusive motions within the spheres of radii *a*_1_ and *a*_2_, respectively (p_0_ + p_1_ + p_2_ = 1). As shown in Figure 5, similar values of the fraction of the “immobile” atoms are obtained from the analyses using both models. Furthermore, the distributions of the radii of spherical spaces in which the atoms undergo diffusive motions appear to be consistent between the two models. Thus, the analyses of the EISF curves using Equations (6) and (7) yield consistent results. Differences between the wtTn-CD and mtTn-CD are observed in the radial distribution of the mobile fractions. In the wtTn-CD, the peak position in the distribution shifts to a larger value, and the width of the distribution is narrower in the high-Ca^2+^ state than in the low-Ca^2+^ state, whereas in the mtTn-CD, the peak position remains similar, but the width widens. Taken together, in the wtTn-CD, the rates of the local motions become faster, and their amplitudes become larger, but their distribution becomes narrower in the high-Ca^2+^ state. On the other hand, in the mtTn-CD, the rates of the local motions do not change, but the distribution of the amplitudes of the local motions becomes wider.

### 3.2. Analysis of the QENS Spectra of αSyn

**Figure 6:**
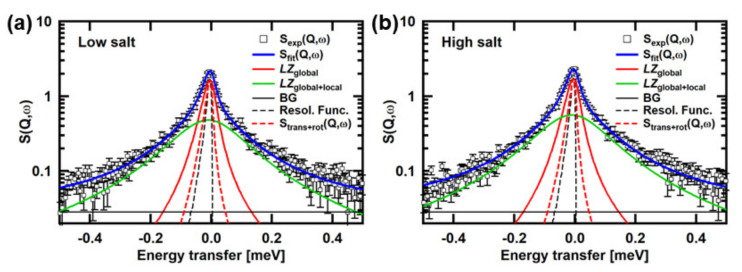
Examples of the QENS spectra. The spectra at *Q* = 1.05 Å^−1^ of αSyn (**a**) in the low-salt state, and (**b**) in the high-salt state, are shown. The fits to the experimental spectra using Equation (1), the simulated spectra of S_trans+rot_(*Q*,*ω*), and the resolution function are also shown. The experimental spectra shown were obtained from the measurements carried out during the study described in [14].

**Figure 7:**
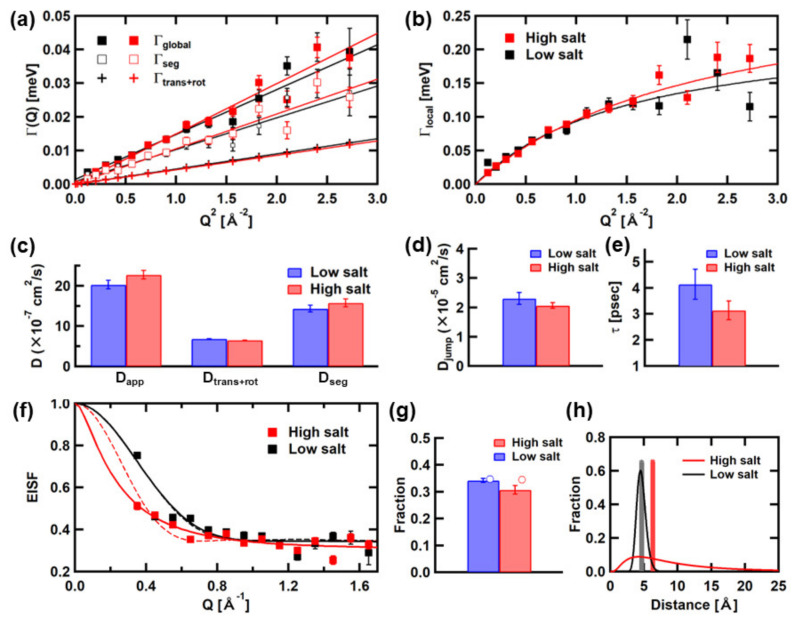
Summary of the analysis of the QENS spectra of αSyn. (**a**) Summary of Q^2^-dependence of the Γ values. Symbols in black and red are those for the high- and low-salt conditions, respectively. (**b**) Summary of Q^2^-dependence of Γ_local_. (**c**) Summary of the diffusion coefficients, D_app_, D_trans+rot_, and D_seg_, obtained from linear fits to the plots in (**a**). (**d**) Summary of D_jump_. (**e**) Summary of the residence time, τ. D_jump_ and τ are obtained from the fits with the equation based on the jump-diffusion model to the plots in (**b**). (**f**) The EISF curves and the results of the fits. Solid lines represent the results of the analysis using Equation (6), and dashed lines represent the results of the analysis based on the diffusion-inside-a-sphere model employed in the previous study [14]. (**g**) A summary of the fraction of the “immobile” atoms. Bars represent the results of the analysis using Equation (6). Open circles represent the results of the analysis based on the diffusion-inside-a-sphere model. (**h**) The radial distributions of the fraction of the “mobile” atoms. Solid lines represent the results of the analysis using Equation (6). Bars represent the results of the analysis based on the diffusion-inside-a-sphere model. Error bars within the symbols are not shown.

The QENS spectra of αSyn in the low- and high-salt conditions at pH 7.4 are re-analyzed. These conditions correspond to the low- and high-propensity states for fibril formation, respectively. Examples of the experimental spectra and the fits to these spectra are shown in Figure 6. Differences in the widths between *LZ*_global_ and S_trans+rot_(*Q,ω*) are more significant than those of the Tn-CD, indicating that the segmental motions are more significant in αSyn. Figure 7a shows the Q^2^-dependences of the widths of *LZ*_global_, the simulated S_trans+rot_(*Q,ω*), and the extracted *L*_seg_(*Q,ω*), and Figure 7c shows the diffusion coefficients calculated as the slopes of the linear fits to these Q^2^-dependences. The values of the diffusion coefficients are summarized in Table 3. It is shown that corresponding to the higher propensity for fibril formation, the diffusion coefficient of the segmental motion, D_seg_, increases, suggesting that the segmental motions are enhanced.

Figure 7b shows Q^2^-dependences of the widths of *LZ*_local_, and the parameters obtained from the fits with Equation (5) to these curves are shown in Figure 7d,e. Whereas the D_jump_ values are within errors, the residence time is lower in the high-salt condition than in the low-salt condition, suggesting that the local motions are faster in the high-salt condition than in the low-salt condition. Figure 7f shows the EISF curves and the fits with Equation (6). The parameters obtained from these fits are shown in Figure 7g,h. Figure 7f–h include the results of the EISF analysis using the diffusion-inside-a-sphere model employed in the previous study [14] for comparison. The model containing only one type of sphere was sufficient to fit the EISF curves of αSyn (Equation (7) but containing only the first and the second terms). As in the case of the Tn-CD, the results of the analyses using both models are consistent (similar values of the fraction of immobile atoms and consistent distribution of the radius of the spherical space in which the atoms undergo diffusive motions). The fraction of the “immobile” atoms decreases slightly, and the distribution of the mobile atoms expands significantly such that more atoms move in larger spaces in the high-salt condition. These results indicate that in the high-propensity state for fibril formation, the rates and the amplitudes of the local motions become faster and larger, respectively. Thus, it is suggested that enhancement of the local motions, as well as the segmental motions, is related to the increased propensity for fibril formation of αSyn in the high-salt condition.

## 4. Discussion

Here it is shown that by the combined analysis of the QENS spectra with the structural data, the segmental motions of the IDR/IDP can be characterized quantitatively in the form of the diffusion coefficient, D_seg_, in addition to the local motions. D_T_ and D_R,_ calculated using the structural models from the SAXS measurements to extract D_seg_, are those of self-diffusion at infinite dilution. These values are known to be significantly reduced in crowded solution: for example, the D_T_ of a globular protein, bovine serum albumin, is reduced to ~0.6 of that at infinite dilution at the volume fraction of 0.1 [28]. Furthermore, hydrodynamic interactions, as well as weak short-range attractions, also reduce these values under crowded conditions [39,40]. The sample solutions for the QENS measurements analyzed here, on the other hand, have the protein concentrations of ~22 mg/mL for the Tn-CD and ~10 mg/mL for αSyn, which correspond to the volume fractions of ~0.016 and ~0.0073, respectively, assuming that the partial specific volume of the proteins is 0.73 cm^3^/g. These solutions are thus considered diluted. It is difficult to estimate how significant these interactions affect the diffusion coefficients under dilute conditions, though a semiempirical equation for hard spheres [41] shows that the short-time self-diffusion coefficients are reduced by 0.971 and 0.987 at the volume fractions of 0.015 and 0.0073, respectively. Thus, although these effects may be weak in dilute solutions, they may still exist. The calculated values of the diffusion coefficients thus indicate upper limits. This implies that the D_seg_ values represent the minimum contribution of the segmental motions.

It is noted that the D_seg_ values obtained are widely different: the values of the Tn-CD are ~2 × 10^−7^ cm^2^/s, whereas those of αSyn are ~15 × 10^−7^ cm^2^/s. These results indicate a variety of segmental motions. The values of the Tn-CD are rather similar to those of the molten-globule state of the proteins than to the unfolded state [15]. This implies that the motions of the IDRs in the Tn-CD are not free diffusive motions but restricted motions, suggesting that significant intramolecular interactions exist. These results are consistent with the results of the SAXS measurements showing that the distributions of the disordered regions in the Tn-CD are not random but rather confined to distinct regions [29].

On the other hand, the D_seg_ values of αSyn are 14.3 ± 0.9 × 10^−7^ cm^2^/s and 15.1 ± 0.8 × 10^−7^ cm^2^/s for the low- and high-salt conditions, respectively. These values are consistent with the values of the intramolecular diffusion coefficients obtained by other techniques [42,43,44], indicating the usefulness of D_seg_. The hydrodynamic radius, R_H_, corresponding to D_seg,_ can be calculated using the Stokes–Einstein equation, D = *k*_B_*T*/6πηR_H_, where *k*_B_ is the Boltzmann constant, η is the viscosity. The values corresponding to D_seg_ of αSyn are 12.6 ± 0.8 Å and 11.9 ± 0.6 Å for the low- and high-salt conditions, respectively. Using the empirical relationship between R_H_ and the number of residues in the polypeptide chain (N) for denatured proteins, R_H_ = 2.21N^0.57^ Å [45], the number of residues in the segments in these conditions is estimated to be 21 ± 3 residues and 19 ± 2 residues, respectively. The difference between the low- and high-salt conditions suggests that the molecule is more flexible in the high-salt condition.

It should be noted that the difference between αSyn in the low- and high-salt conditions may arise from the charge-screening effect that could affect the values of self-diffusion coefficients under the high-salt condition. Whereas this effect appears to be minor in the Tn-CD because the difference in the low- and high-Ca^2+^ states is 5 mM CaCl_2_, it could be significant for αSyn because the high-salt condition contains 150 mM NaCl. However, a simulation study of charged and neutral spheres [46] revealed that the self-diffusion coefficients of the charged sphere at finite volume fractions are smaller than those at infinite dilution and that the addition of salt further reduces these values. These findings suggest that charge-screening by the addition of salt to charged particles reduces the diffusion coefficients of the particles, and thus in the case of αSyn, the inclusion of the charge-screening affects the direction of amplifying the difference between the low- and high-salt conditions. The conclusions obtained from the current analysis are thus valid if the self-diffusion coefficients from the structural data are used.

These segmental motions of αSyn appear to be in concert with the local motions. As shown in Figure 7e,h, the rates of the local motions are enhanced, and the distribution of the amplitudes of the local motions is widened such that the motions with larger amplitudes occur in the high-salt condition. Considering the fact that the high-salt condition corresponds to a high-propensity state for fibril formation, enhancement of the local motions as well as the segmental motions is important in starting the process of fibril formation.

The relationship between the segmental motions and the local motions is different between the wtTn-CD and the mtTn-CD. In the wtTn-CD, the segmental motions are enhanced in the high-Ca^2+^ state, and the rates of the local motions are enhanced as well, but the distribution of the amplitudes of the local motions is somewhat narrower though the peak position of the distribution shifts to a larger value. On the other hand, in the mtTn-CD, the segmental motions are similar to those in the wtTn-CD in the low-Ca^2+^ state but significantly suppressed in the high-Ca^2+^ state. The rates of the local motions remain similar but at the level of the wtTn-CD in the high-Ca^2+^ state. The distribution of the amplitudes of the local motions is wider in the high-Ca^2+^ state, containing the motions of larger amplitudes than in the low-Ca^2+^ state. Such differences are attributable to destabilized structures of the mtTn-CD by disruption of the hydrogen-bond network by the mutation, as discussed in previous studies [13,24]. In addition, the different behavior of the segmental motions implies that intramolecular interactions are changed by the mutation. Suppression of the segmental motions of the mtTn-CD in the high-Ca^2+^ state implies increased intramolecular interactions, which is consistent with the observation by the SAXS measurements [29] that the disordered regions in TnT and TnI, TnT_183–223_ and TnI_137–210_, move closer to the central region in the Tn-CD. TnT_183–223_ contains a linker connecting the core region and the region that binds to and interacts with Tm, which controls the interaction between actin and myosin [25]. TnI_137–210_ is involved with the regulatory system by Ca^2+^ of Tn [25]. Changes in the behavior of these regions should directly affect the function of Tn. The observation that the significant behavioral changes by mutation occur in the high-Ca^2+^ state is consistent with the fact that the functional aberration of this mutant occurs in the high-Ca^2+^ state [24].

Recent QENS studies on a denatured protein [47] and an IDP [48] revealed that the internal dynamics of the denatured proteins exhibit distinct behavior due to an inherent dynamical heterogeneity, which depends on the degrees of the unfolding of the proteins. Such changes in the dynamical heterogeneity could be related to the abnormal behavior associated with the pathogenesis of diseases. D_seg,_ in the analysis described here, could detect such changes and, as demonstrated here, is useful for analyzing the behavior possibly related to the pathogenesis of the diseases.

## 5. Conclusions

Here, it is shown that the parameter, D_seg_, which describes the segmental motions of the disordered regions in proteins, can be extracted by the combined analysis of QENS spectra with structural data, and this parameter is useful for describing the dynamical behavior of the proteins containing the disordered regions as well as the IDPs. In particular, it is demonstrated through the discussion on the two examples of the disease-related proteins that characterization of the segmental motions, as well as the local motions, is useful for discussing in detail how the changes in the dynamical behavior due to disease-related mutation and/or environmental changes could be related to the functional and/or behavioral aberration of these proteins. The insights obtained could eventually contribute to developing a therapeutic strategy for these diseases.

## Figures and Tables

**Table 1:** Summary of the diffusion coefficients calculated from the structural models.

	wtTn-CD Low Ca^2+^	wtTn-CD High Ca^2+^	mtTn-CD Low Ca^2+^	mtTn-CD High Ca^2+^	αSyn Low Salt	αSyn High Salt
D_T_ (×10^−7^ cm^2^/s)	4.64 ± 0.01	4.68 ± 0.02	4.79 ± 0.02	4.87 ± 0.02	5.59 ± 0.03	4.70 ± 0.04
D_R_ (×10^6^ 1/s)	1.62 ± 0.13	1.64 ± 0.17	1.76 ± 0.16	1.86 ± 0.18	2.54 ± 0.03	1.63 ± 0.06

**Table 2:** Summary of the diffusion coefficients of the Tn-CD.

	D_app_ (×10^−7^ cm^2^/s)	D_trans+rot_ (×10^−7^ cm^2^/s)	D_seg_ (×10^−7^ cm^2^/s)
wtTn-CD, Low-Ca^2+^ state	8.02 ± 0.15	5.92 ± 0.02	2.28 ± 0.16
wtTn-CD, High-Ca^2+^ state	8.24 ± 0.14	5.99 ± 0.02	2.43 ± 0.15
mtTn-CD, Low-Ca^2+^ state	8.24 ± 0.14	6.09 ± 0.02	2.39 ± 0.16
mtTn-CD, High-Ca^2+^ state	8.18 ± 0.14	6.22 ± 0.02	2.02 ± 0.15

**Table 3:** Summary of the diffusion coefficients of αSyn.

	D_app_ (×10^−7^ cm^2^/s)	D_trans+rot_ (×10^−7^ cm^2^/s)	D_seg_ (×10^−7^ cm^2^/s)
Low salt	20.3 ± 1.1	6.88 ± 0.01	14.3 ± 0.9
High salt	21.7 ± 0.9	6.49 ± 0.02	15.1 ± 0.8

## Data Availability

The data analyzed here are available upon request to the author.
